# Post-Treatment MRI Features on First Follow-Up Imaging in Diffuse Gliomas After Near-Total Resection: A Real-World Exploratory Cohort Study

**DOI:** 10.3390/medicina62061136

**Published:** 2026-06-10

**Authors:** Teodor Cristian Blidaru, Dan Mitrea, Nicolae Dragoș Garofil, Ileana Ruxandra Spătaru, Raluca Maria Marin, Marius Cristian Zaharia, Sebastian Romeo Pintilie, Stefan Strilciuc, Ioana Raluca Papacocea

**Affiliations:** 1 Faculty of Medicine, Carol Davila University of Medicine and Pharmacy, Dionisie Lupu 37, 020021 Bucharest, Romania; 2 Neuroaxis Neurology Clinic, 040215 Bucharest, Romania; 3 Faculty of Medicine, Iuliu Hatieganu University of Medicine and Pharmacy, 400012 Cluj-Napoca, Romania; 4 Department of Genomics, Medfuture Institute for Biomedical Research, Iuliu Hatieganu University of Medicine and Pharmacy, 400349 Cluj-Napoca, Romania

**Keywords:** diffuse glioma, magnetic resonance imaging, postoperative imaging, residual tumor, near-total resection, real-world cohort, brain tumor recurrence, neuro-oncology, imaging biomarkers

## Abstract

*Background and Objectives*: Magnetic resonance imaging (MRI) is essential for monitoring patients with diffuse gliomas after surgery and adjuvant therapy. However, the prognostic significance of imaging findings observed on the first post-treatment MRI remains incompletely defined in routine clinical practice. This study aimed to evaluate whether MRI features identified on the first post-treatment examination are associated with recurrence-free survival after near-total resection (NTR), defined for this real-world cohort as ≥80% volumetric tumor removal (which differs from the RANO Resect Group definition of GTR). *Materials and Methods*: Consecutive adult patients with histologically confirmed diffuse gliomas diagnosed between 2021 and 2024 were screened for eligibility (*n* = 283) and classified according to the 2021 WHO Classification of Tumors of the Central Nervous System. The first post-treatment MRI was defined as the earliest follow-up examination performed after completion of all initially planned therapy (surgery alone in patients without indication for adjuvant therapy; post-radiotherapy ± chemotherapy in the remaining patients), and not the immediate postoperative scan. Patients with available first post-treatment MRI were included (*n* = 149), resulting in a final cohort of 139 cases after exclusion of outliers. The evaluated MRI corresponded to the first follow-up examination after treatment (mean interval ~120 days after surgery). Logistic regression models were used to assess associations between post-treatment MRI features and recurrence-free survival at one and two years after NTR, with exploratory analyses for reoperation and reirradiation. *Results*: Residual tumor identified on the first post-treatment MRI was associated with lower odds of recurrence-free survival (RFS) at one and two years after NTR. Postoperative functional status also demonstrated an independent association with tumor control. Other imaging variables showed associations in univariable analyses but did not remain independent predictors after adjustment. Exploratory analyses of reoperation and reirradiation suggested additional clinical influences, including patient age. *Conclusions*: In this real-world cohort with heterogeneous tumor subtypes and treatment regimens, residual tumor on first follow-up MRI was the most consistent imaging correlate of reduced RFS, alongside postoperative functional status. These hypothesis-generating findings should be validated in stratified, prospective cohorts.

## 1. Introduction

Diffuse gliomas represent the predominant category of primary malignant tumors of the central nervous system and remain associated with poor clinical outcomes despite advances in multimodal therapy [1,2]. Contemporary classification systems, particularly the 2021 World Health Organization (WHO) framework, integrate histopathological and molecular parameters, reflecting the biological heterogeneity and variable clinical behavior of these tumors [3,4]. High-grade gliomas are especially characterized by aggressive growth, diffuse infiltration of the surrounding brain parenchyma, and a high propensity for recurrence [2,5].

Near-total resection (NTR)—a contemporary surgical paradigm that encompasses complete resection of the contrast-enhancing component (historically referred to as gross total resection, GTR) in high-grade gliomas and supramaximal volumetric removal where feasible in lower-grade tumors—constitutes the surgical foundation of treatment, typically followed by radiotherapy and systemic chemotherapy [2]. The extent of tumor removal is a well-established determinant of prognosis, with GTR associated with improved overall and progression-free survival [6,7]. However, due to the infiltrative nature of gliomas, microscopic tumor spread frequently extends beyond radiologically visible margins, contributing to the high rate of local recurrence even after apparently complete resection [8,9].

Magnetic resonance imaging (MRI) plays a central role in the management of glioma patients, providing essential information for diagnosis, treatment planning, and longitudinal monitoring [10,11]. In the postoperative setting, MRI is critical for assessing the extent of resection, establishing a baseline for follow-up, and detecting early disease progression [12,13]. The integration of advanced imaging techniques, including diffusion and perfusion sequences, has further enhanced the ability of MRI to characterize tumor biology and treatment response [14,15].

Despite these advances, interpretation of imaging findings in the early post-treatment period remains challenging. Radiological appearances at this stage often reflect a complex interplay between residual tumor and treatment-related changes, including inflammatory responses, vascular permeability alterations, and radiation-induced effects [16,17]. This overlap reduces diagnostic specificity and complicates the differentiation between true tumor persistence and therapy-related changes [18]. In addition, phenomena such as pseudoprogression and treatment-related contrast enhancement further limit the reliability of early imaging assessment and pseudoresponse, particularly in the post-radiotherapy and post-antiangiogenic setting [19].

The first MRI examination performed after completion of initial treatment represents a critical time point in the disease course. It establishes the reference baseline for subsequent follow-up and may directly influence clinical decision-making [12,13]. However, the prognostic significance of imaging findings identified at this stage remains incompletely defined. Although the prognostic implications of these findings may differ across glioma subtypes, the conventional MRI features evaluated at this time point share a common interpretive framework rooted in routine clinical workflow.

To date, most imaging studies in gliomas have focused on preoperative biomarkers, advanced radiomics approaches, or long-term survival prediction models [20,21]. Although these approaches have improved understanding of tumor heterogeneity, their applicability in routine clinical practice is often limited. In contrast, conventional MRI features observed on the first post-treatment examination, readily available in daily practice, have been less extensively studied.

Emerging evidence suggests that certain imaging features, particularly the presence of residual contrast-enhancing tumor, may be associated with adverse outcomes and reduced survival [7,8]. However, these findings are not consistently reported, and their independent prognostic value in real-world clinical settings remains uncertain. Furthermore, the interaction between early imaging findings and clinical factors, such as postoperative functional status and patient characteristics, has not been fully clarified [4].

Against this background, the present study aimed to exploratorily evaluate the association between MRI features identified on the first post-treatment examination and recurrence-free survival at one and two years following near-total resection in patients with diffuse gliomas. In addition, exploratory analyses were conducted to assess the relationship between early imaging findings and subsequent clinical interventions, including reoperation and reirradiation. By focusing on routinely available imaging parameters within a real-world cohort, this study seeks to clarify the clinical relevance of early post-treatment MRI and to identify practical markers of recurrence risk that may inform individualized follow-up strategies. Given the observational, single-center design and biological heterogeneity of the cohort, findings are intended to be hypothesis-generating and to inform the design of future stratified, prospective studies.

## 2. Materials and Methods

### 2.1. Study Design

This single-center retrospective observational cohort study with consecutive patient inclusion was designed and conducted at a tertiary neuro-oncology center in Romania. Reporting was performed in accordance with the Strengthening the Reporting of Observational Studies in Epidemiology guidelines for observational studies [22].

The study evaluated the association between first post-treatment magnetic resonance imaging (MRI) features and recurrence-free survival after NTR in adult patients with diffuse gliomas. Consecutive patients diagnosed with histologically confirmed diffuse gliomas between 2021 and 2024 were screened for eligibility using an institutional database.

The first post-treatment MRI was defined as the first follow-up examination performed after completion of all initial therapeutic interventions, including surgery with or without adjuvant radiotherapy and/or chemotherapy, rather than the immediate postoperative scan. This definition is consistent with clinical practice recommendations emphasizing the importance of follow-up imaging after completion of primary therapy [2,11]. Depending on the individual therapeutic course, this MRI was obtained either after a short postoperative recovery period in patients who did not require adjuvant treatment or, more commonly, after radiotherapy and/or chemotherapy. For analytical purposes, this examination was defined as: (a) the first MRI performed ≥30 days after surgery in patients managed with surgery alone (typically lower-grade IDH-mutant tumors not requiring adjuvant therapy at presentation); or (b) the first post-completion-of-radiotherapy MRI (typically performed 4–6 weeks after the end of radiotherapy) in patients undergoing adjuvant therapy. We acknowledge that this composite definition introduces variability in the timing relative to surgery, which is addressed in the Limitations.

The primary objective was to assess whether post-treatment MRI features were associated with recurrence-free survival at one and two years after NTR. Secondary exploratory objectives included the evaluation of associations between post-treatment MRI findings and the need for reoperation or reirradiation during follow-up.

The study was conducted in accordance with the Declaration of Helsinki [23] and approved by the Research Ethics Subcommittee of Carol Davila University of Medicine and Pharmacy, Bucharest, Romania (registration number 35495/04.12.2025).

### 2.2. Study Population

Consecutive adult patients diagnosed with histologically confirmed diffuse gliomas between January 2021 and December 2024 were screened for eligibility using an institutional neuro-oncology imaging database (*n* = 283). Tumors were classified according to the 2021 World Health Organization (WHO) Classification of Tumors of the Central Nervous System [3].

Clinical and demographic variables were extracted from institutional medical records and the imaging database. Patients were included if they had available post-treatment MRI examinations performed after the completion of the initial treatment phase. Only patients with sufficient follow-up data allowing evaluation of recurrence-free survival were retained for outcome analyses.

**Figure 1:**
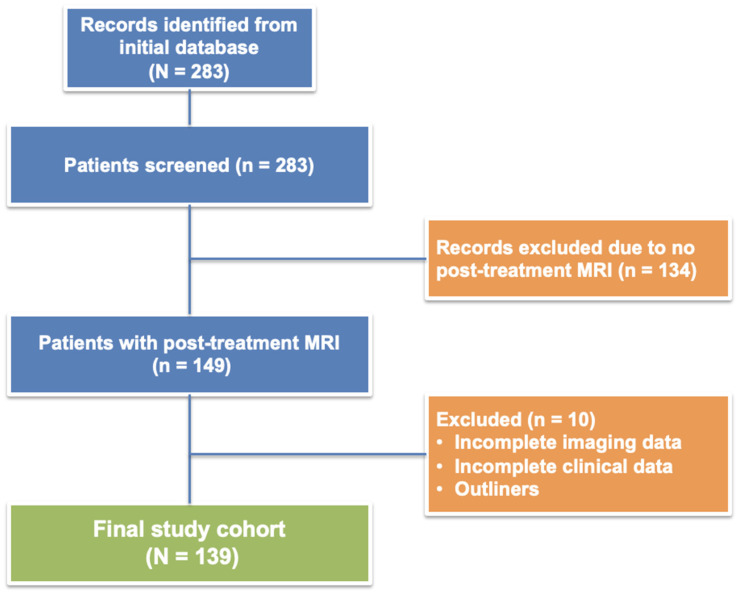
Patients’ selection flow.

Due to the retrospective nature of the study and variable data availability, the effective sample size differed across analyses. The initial cohort consisted of 283 screened patients, of whom 149 had available post-treatment MRI, and 139 were included in the final descriptive cohort after exclusion of outliers and incomplete data. For regression analyses, only cases with complete data for the variables included in each model were analyzed (complete-case analysis), resulting in smaller effective sample sizes that are reported for each model and table. The patient selection process is illustrated in the STROBE flow diagram (Figure 1).

### 2.3. MRI Evaluation

In the study cohort, this imaging was performed at a mean interval of approximately 120 days after surgery. Standard brain tumor MRI protocols included conventional anatomical sequences routinely used in clinical practice, such as T1-weighted imaging (before and after gadolinium-based contrast administration), T2-weighted imaging, fluid-attenuated inversion recovery (FLAIR) and SWI sequences. Additional sequences were acquired according to clinical indications.

Where DSC perfusion was acquired, rCBV was assessed qualitatively by the reporting neuroradiologist using visual comparison against contralateral normal-appearing white matter, and categorized as highly increased, increased, normal, or decreased. Acquisition parameters for the conventional and advanced MRI sequences varied across the 2021–2024 accrual window, reflecting routine clinical practice across multiple scanner generations. This protocol heterogeneity is acknowledged as a limitation.

The interval between surgery and the first post-treatment MRI was non-uniform across the cohort, reflecting the composite definition adopted (post-surgery-only vs. post-radiotherapy or chemoradiotherapy). The mean interval of approximately 120 days reported here is an estimate derived from retrospective review of the clinical chart record. This is acknowledged as a limitation and motivates harmonized capture of MRI timing in future prospective work.

Post-treatment MRI examinations were reviewed, and relevant imaging variables were extracted from the institutional imaging database. The analyzed variables focused primarily on structural imaging features that could reflect residual disease, treatment-related changes, or early tumor progression patterns.

Imaging variables included the presence or absence of residual tumor, contrast-enhancing components, and other structural abnormalities identified on the first post-treatment MRI. Postoperative hemorrhage was defined as any susceptibility-related signal on SWI sequences, including small or clinically insignificant findings, which may explain its high observed prevalence. These variables were treated as independent predictors in the statistical analyses and were evaluated for potential associations with recurrence-free survival and other clinical outcomes.

**Figure 2:**
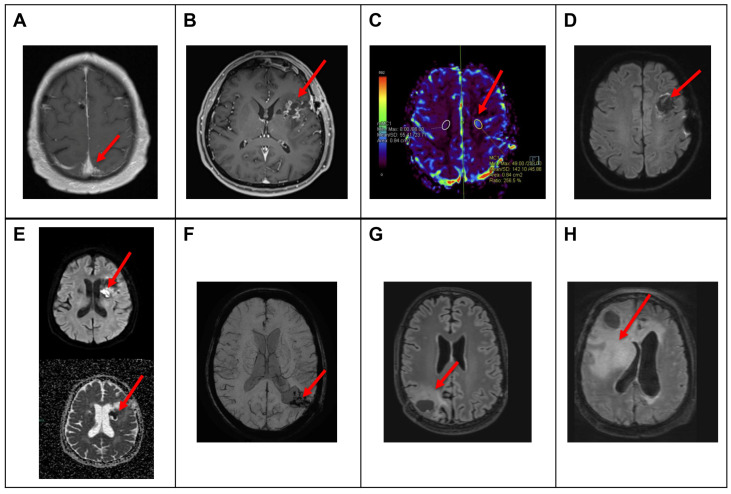
Representative post-treatment MRI features evaluated in the study cohort. Red arrows in all panels indicate the imaging feature described in the corresponding panel label. Note: (**A**) Postoperative residual tumor adjacent to the resection cavity on contrast-enhanced T1-weighted imaging. (**B**) Residual tumor showing contrast enhancement along the surgical cavity wall. (**C**) Increased relative cerebral blood volume (rCBV) on perfusion imaging. (**D**) Diffusion restriction within tumor tissue on diffusion-weighted imaging. (**E**) Postoperative infarction adjacent to the surgical cavity. (**F**) Postoperative hemorrhage visible on susceptibility-weighted imaging. (**G**) Peritumoral edema on FLAIR imaging. (**H**) Midline shift secondary to postoperative mass effect.

**Figure 3:**
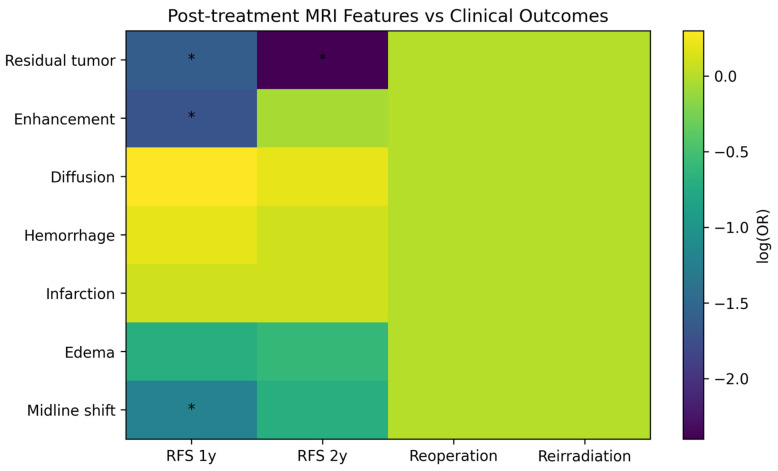
Integrated overview of post-treatment MRI predictors across clinical outcomes. Asterisks (*) indicate cells reaching the conventional significance threshold (*p* < 0.05) in the corresponding regression model.

Nine imaging features were evaluated on the first post-treatment MRI and are reported consistently across the Methods, Figure 2, Figure 3 heatmap, the regression tables, and Supplementary Materials Tables S1–S7: (1) residual tumor—any nodular T1 contrast-enhancing tissue along the resection cavity wall, or any T2/FLAIR hyperintense mass-like tissue beyond expected post-surgical signal in non-enhancing tumors; (2) residual tumor contrast enhancement—presence or absence of contrast enhancement of any residual tumor tissue identified; (3) location of residual tumor—categorized as adjacent cortex, deep margin, cavity wall, or multifocal; (4) relative cerebral blood volume (rCBV) trend on DSC perfusion—categorized as highly increased, increased, normal, or decreased as detailed above; (5) diffusion restriction—restricted diffusion on DWI within the residual tumor or peri-cavity tissue beyond expected post-surgical change; (6) postoperative infarction—restricted diffusion in vascular territory adjacent to the resection cavity persisting beyond 7 days post-surgery; (7) postoperative hemorrhage—any susceptibility-related hypointensity on SWI within or adjacent to the resection cavity; (8) peritumoral edema—T2/FLAIR hyperintensity surrounding the resection cavity beyond expected post-surgical changes; (9) midline shift—measured at the level of the septum pellucidum, considered present if ≥3 mm (Figure 2). Variables 4 and 5 (rCBV trend and diffusion restriction) were available only in the subset of patients who underwent advanced imaging; this differential availability accounts for the smaller effective sample size of the corresponding multivariable models. Ambiguous findings (e.g., possible early progression vs. pseudoprogression) were adjudicated by consensus during routine multidisciplinary tumor board review.

All imaging assessments were performed as part of routine clinical evaluation and subsequently curated in the institutional research database used for this study. All imaging interpretations were issued by board-certified neuroradiologists who had full access to the clinical and surgical history of each patient, as required by routine clinical workflow; formal blinding to outcome was not feasible in this retrospective design. Because recurrence ascertainment likewise relied on multidisciplinary review of the same imaging stream, this design introduces a potential for observer bias and for incorporation bias (the use of imaging information both in the exposure and in the outcome definition). This is intrinsic to a real-world retrospective cohort built from routine clinical reports and is acknowledged explicitly in the Limitations.

### 2.4. Variables and Outcomes

The primary dependent variables were recurrence-free survival (RFS) at one and two years after near-total resection (NTR). NTR was operationally defined for this real-world cohort as ≥80% volumetric resection of the contrast-enhancing component (in high-grade gliomas) or of the T2/FLAIR-hyperintense tumor volume (in non-enhancing lower-grade gliomas), as assessed on the immediate postoperative MRI by the operating neurosurgical and neuroradiology team. We acknowledge that this composite, volumetric threshold differs from stricter definitions of GTR (e.g., complete resection of the contrast-enhancing component as recommended by the RANO Resect group [24]), and our use is intended to reflect a practical near-total resection cohort rather than to advance a new definition. The implications of this operational definition are addressed in the Limitations.

Residual tumor was assessed on post-treatment MRI performed at a mean interval of approximately 120 days after surgery and may reflect post-treatment changes.

Independent variables consisted of imaging features identified on the first post-treatment MRI examination, primarily reflecting residual tumor or other structural abnormalities potentially associated with tumor progression.

Clinical covariates included patient age, sex, tumor laterality, tumor grade, histological tumor type, extent of resection, and postoperative functional status assessed using the Karnofsky Performance Status (KPS). These variables were evaluated for potential associations with recurrence-related outcomes using regression-based statistical models.

Recurrence was defined according to RANO 2.0 criteria adapted for clinical practice [12]: (i) for high-grade gliomas, unequivocal new or progressive contrast-enhancing tissue ≥10 mm in maximum diameter, or unequivocal increase in non-enhancing T2/FLAIR signal in non-enhancing tumors, on serial follow-up MRI, persisting on confirmatory imaging at least 4 weeks later, in the absence of an alternative explanation (treatment-related changes, hemorrhage, ischemia); (ii) for lower-grade gliomas, similar criteria with longer confirmatory intervals (8–12 weeks) per institutional practice. All putative recurrence events were adjudicated retrospectively by consensus of the neuro-oncology multidisciplinary team based on serial imaging review and, when available, histopathological confirmation at re-resection. Pseudoprogression and treatment-related changes were specifically considered in the differential of any new enhancing lesion appearing within 12 weeks of completion of radiotherapy [17].

### 2.5. Statistical Analysis

Statistical analysis was performed using Jamovi software (version 2.6.26, jamovi.org, The jamovi project, Sydney, Australia). Qualitative variable categories with very low frequencies (<15 observations) were eliminated or merged where possible in order to improve the robustness of the statistical analysis.

Univariable logistic regression models were used to investigate potential predictors of recurrence-related outcomes, including recurrence-free survival at one year and two years after near-total resection (NTR), as well as the exploratory outcomes of reoperation and reirradiation during follow-up.

Variables exhibiting a Wald test *p*-value < 0.20 in the univariable analysis were considered for inclusion in the multivariable models. In the case of variables with more than two categories, the likelihood ratio test *p*-value was used instead. In addition, the clinically relevant variable sex was included in the candidate variables considered for the multivariable models.

Variables or categories exhibiting quasi-complete or complete separation were excluded from the analysis. Furthermore, variables with fewer than 50 observations in the entire sample were not included in the multivariable models even if they were statistically significant in the univariable analysis.

For each univariable model, odds ratios (OR) with corresponding confidence intervals were calculated. In the multivariable logistic regression models, adjusted odds ratios (aOR) were computed for each included variable. Model performance was evaluated using the Akaike Information Criterion (AIC), McFadden’s R^2^, and the overall model *p*-value. A two-sided *p*-value < 0.05 was considered statistically significant.

All multivariable models were estimated by complete-case analysis. The effective sample size and the number of events at each landmark are reported beneath the corresponding multivariable table: 1-year RFS (*n* = 93, 31 events), 2-year RFS (*n* = 93, 34 events), reoperation (*n* = 44, 14 events), and reirradiation (*n* = 41, 18 events). Missing data were concentrated in two areas: advanced imaging variables (rCBV trend on DSC perfusion, diffusion restriction), available only in a subset of patients reflecting non-uniform acquisition of advanced sequences across the 2021–2024 accrual window; and exact-date recurrence timing, imprecise in a subset of cases adjudicated retrospectively from the multidisciplinary tumor board record. The low events-per-variable ratio in the exploratory reoperation and reirradiation models carries a non-trivial risk of overfitting, and the corresponding findings are therefore reported as hypothesis-generating only. The heatmap visualization (Figure 3) was generated in Python (version 3.11; Python Software Foundation, Beaverton, OR, USA; python.org) using matplotlib and seaborn libraries; the plotting code was developed with the assistance of a large language model (ChatGPT, OpenAI, San Francisco, CA, USA; GPT-5 model; chatgpt.com), and all generated outputs were reviewed, validated and corrected by the authors prior to inclusion. No artificial intelligence tools contributed to study design, data analysis, statistical modeling, interpretation of results, or formulation of conclusions; AI tools were limited to language editing assistance (see Acknowledgments) and to code-generation assistance for visualization purposes.

Figure 3 Integrated heatmap of post-treatment MRI predictors across clinical outcomes. Rows represent the imaging variables evaluated on the first post-treatment MRI; columns represent the four assessed outcomes (1-year RFS, 2-year RFS, reoperation, reirradiation). Color intensity is proportional to the magnitude of log OR, with the intensity scale displayed in the lower-right legend (range −2 to +2). Cells reaching the conventional significance threshold (*p* < 0.05) in the corresponding multivariable model are marked with an asterisk (*); unmarked cells should be interpreted as non-significant trends, displayed for descriptive comparison only and to be read as hypothesis-generating rather than as evidence of association. Variables that could not be estimated in a given multivariable model (owing to quasi-complete separation, insufficient events, or unavailable advanced-sequence data) are omitted from the corresponding column. Underlying numerical values, exact *p*-values, and confidence intervals are provided in Supplementary Tables S3–S5; the heatmap is intended as a visual summary subordinated to those numerical tables.

## 3. Results

### 3.1. Patient Characteristics

A total of 139 patients with diffuse gliomas and available post-treatment MRI examinations were included in the final analysis, as presented in Table 1. The cohort included 77 female (55.4%) and 62 male (44.6%) patients. Glioblastoma was the most frequent histological subtype (57.6%), followed by astrocytoma (30.2%) and oligodendroglioma (12.2%). Most tumors were WHO grade 4 (61.9%).

Regarding postoperative functional status, 52.2% of patients had a Karnofsky Performance Status (KPS) of 100, while 32.8% had KPS 90.

On first post-treatment MRI, residual tumor was identified in 82.6% of cases, with contrast enhancement present in 77.2% of patients. Diffusion restriction was observed in 34.4% of cases, postoperative infarction in 8.8%, and midline shift in 22.6% of patients.

The high prevalence of residual tumor on the first post-treatment MRI (82.6%) is consistent with the near-total resection definition adopted in this study (≥80% volumetric removal, rather than complete resection of the contrast-enhancing component as in the RANO Resect Group definition of GTR). Three factors jointly account for this prevalence: (i) the operational ≥80% volumetric threshold by construction admits patients with up to ~20% residual tumor volume; (ii) the residual-tumor definition explicitly includes T2/FLAIR hyperintense tissue beyond expected post-surgical signal in non-enhancing (predominantly lower-grade IDH-mutant) tumors, which would not be counted under a contrast-enhancing-only definition; and (iii) the first post-treatment MRI was performed at a mean interval of approximately 120 days, by which time treatment-related changes (post-operative inflammation, early post-radiotherapy enhancement, pseudoprogression) may contribute to apparent residual signal in a subset of cases. The 82.6% figure should therefore not be read as a measure of surgical performance but as a structural characteristic of this real-world near-total resection cohort.

Within the analytic cohort, molecular data were available for 91.4% of patients for IDH mutation status (127/139), 89.9% for KI-67 proliferation index (125/139), and 78.4% for ATRX nuclear expression (109/139); 1p/19q codeletion was available for 24.5% (34/139), and other molecular markers (TERT promoter mutation, EGFR amplification, p16/CDKN2A, CDKN2A/B homozygous deletion, H3 K27M) were available for fewer than 11% of patients. Among the 127 patients with known IDH status, 54 (42.5%) were IDH-mutant and 73 (57.5%) were IDH-wildtype, with the expected distribution across WHO 2021 subtypes (1.4% IDH-mutant in glioblastoma, 90.2% in astrocytoma, 100% in oligodendroglioma). Detailed descriptive distributions of molecular markers and stratified RFS estimates are provided in the Supplementary Tables S6 and S7.

### 3.2. Predictors of 1-Year Recurrence-Free Survival (RFS)

In univariable logistic regression analysis, several variables were associated with RFS at one year after NTR, as presented in Table 2. Higher postoperative functional status was significantly associated with improved recurrence-free outcomes, with patients presenting a postoperative KPS of 100 showing increased odds of remaining recurrence-free at one year (OR 4.16, 95% CI 1.30–13.36, *p* = 0.016). Imaging variables associated with lower odds of RFS in univariable analysis included the presence of residual tumor, residual tumor contrast enhancement, and midline shift. In the multivariable model, postoperative KPS of 100 remained an independent predictor of RFS (aOR 4.18, 95% CI 1.01–17.18, *p* = 0.047), while the presence of residual tumor showed a borderline association (aOR 0.10, 95% CI 0.01–1.04, *p* = 0.054).

Descriptive 1- and 2-year RFS estimates stratified by histology, WHO 2021 grade, and IDH status are reported in Supplementary Table S7. The expected biological gradient was observed: 1-year RFS was 60.3% in glioblastoma (38/63), 82.4% in astrocytoma (28/34), and 100% in oligodendroglioma (15/15), with grade-stratified estimates concordant with the histology-stratified pattern; within the IDH-known subset (*n* = 127), IDH-mutant cases displayed higher RFS proportions than IDH-wildtype cases at both landmarks. These stratified estimates are descriptive; subtype-stratified multivariable modeling was not performed owing to limited subgroup event counts.

### 3.3. Predictors of 2-Year Recurrence-Free Survival (RFS)

In univariable analysis, higher postoperative functional status and absence of residual tumor were associated with RFS at two years after NTR, as presented in Table 3. In multivariable analysis, postoperative KPS of 100 remained a significant independent predictor of favorable outcomes (aOR 4.94, 95% CI 1.17–20.79, *p* = 0.030). In addition, the presence of residual tumor was independently associated with lower odds of remaining recurrence-free at two years (aOR 0.09, 95% CI 0.009–0.92, *p* = 0.042). Other imaging variables, including residual tumor enhancement and midline shift, did not retain statistical significance in the multivariable model. The full regression analyses are provided in Supplementary Table S3.

### 3.4. Factors Associated with Additional Treatment During Follow-Up (Reoperation or Reirradiation)

Exploratory regression analyses were also performed to evaluate potential associations of clinical and imaging variables with subsequent surgical reintervention and postoperative re-irradiation. Owing to the limited number of events at these endpoints (reoperation *n* = 44 with 14 events; reirradiation *n* = 41 with 18 events) and the corresponding low events-per-variable ratio in the multivariable models, these analyses are reported as only hypothesis-generating and should not be interpreted as identifying independent predictors of either outcome. Within these exploratory analyses, increasing age at diagnosis showed a consistent association with a lower likelihood of both reoperation (aOR 0.85, 95% CI 0.75–0.95) and re-irradiation (aOR 0.88, 95% CI 0.79–0.98) in the multivariable models. Given the low events-per-variable ratio, this association—although directionally consistent across both outcomes—is reported descriptively and is not interpreted as an independent predictor. No postoperative imaging variables demonstrated consistent or robust associations with either subsequent reoperation or re-irradiation after adjustment for clinical covariates. Some imaging features showed non-significant trends in univariable analyses, but none persisted in the multivariable models, most likely reflecting limited statistical power and small event counts rather than the absence of any underlying association.

Taken together, these exploratory analyses indicate that the clinical decisions to proceed with reoperation or re-irradiation in this cohort were not strongly aligned with any single first post-treatment MRI feature after adjustment, in contrast to the consistent association of residual tumor and postoperative functional status with recurrence-free outcomes described in Section 3.2 and Section 3.3. The reoperation and re-irradiation findings should therefore be regarded as hypothesis-generating only.

## 4. Discussion

The present study reports that, in a real-world cohort of adult diffuse gliomas, certain MRI findings on the first post-treatment examination are associated with recurrence-free outcomes. Among the imaging variables evaluated, the presence of residual tumor was the most consistent imaging correlate of reduced RFS at both one and two years following near-total resection, broadly concordant with prior reports on extent-of-resection effects in glioblastoma [7]. In parallel, postoperative functional status, indexed by the Karnofsky Performance Status (KPS), showed an independent association with recurrence-free outcomes [6]. These observations are consistent with the well-recognized prognostic weight of residual tumor burden and functional reserve in glioma practice and should be interpreted as associations within a heterogeneous exploratory cohort rather than as evidence of independent predictive validity.

The association between residual tumor and unfavorable outcomes likely reflects the presence of persistent viable tumor tissue, despite the high resection extent achieved in this cohort. This finding should also be interpreted in the context of the infiltrative growth pattern of diffuse gliomas [5], in which tumor cells frequently extend beyond radiologically visible margins and contribute to early local recurrence [8]. Importantly, in the present study, the evaluated MRI did not correspond to the immediate postoperative examination, but rather to the first follow-up imaging performed after completion of initial treatment, at a mean interval of approximately 120 days. This observation is concordant with recent reports emphasizing the prognostic weight of residual contrast-enhancing tissue on early follow-up imaging across heterogeneous glioma cohorts [25], although the magnitude and consistency of this association vary across studies depending on imaging timing, definition of residual tumor, and stratification by molecular subtype.

Contrast enhancement of the residual tumor alone did not retain independent prognostic significance in multivariable models [16]. This observation is consistent with the limited specificity of enhancement in the post-treatment setting [14], where blood–brain barrier disruption, inflammation, and treatment-related changes may produce imaging patterns that overlap with viable tumor tissue [26]. These findings support the notion that enhancement should not be interpreted in isolation, but rather in conjunction with additional imaging and clinical parameters. This is also concordant with the recognized challenge of distinguishing residual viable tumor from post-radiotherapy reactive enhancement during the 12-week window after completion of radiotherapy, where conventional MRI alone has limited specificity. Recent clinical predictor analyses for pseudoprogression [17] further illustrate the multifactorial nature of this differential, underscoring the need to interpret enhancement in the context of clinical trajectory and serial imaging rather than as an isolated finding.

Midline shift was associated with RFS in univariable analysis but did not remain significant after adjustment. This suggests that midline shift may primarily reflect overall tumor burden or mass effect rather than independently influencing disease progression. Similarly, other structural imaging variables did not retain significance in multivariable models, highlighting the dominant prognostic role of residual tumor burden and functional status in this cohort.

From a clinical perspective, the first post-treatment MRI is a natural decision point in routine glioma follow-up, at which the multidisciplinary team integrates imaging, surgical, and functional information [2,13]. The association between residual tumor on this examination and subsequent recurrence supports careful description of residual disease in routine reporting [7,24] and may inform follow-up intensity at the level of the individual patient [2]. The results presented here, however, do not establish that residual tumor on the first post-treatment MRI is an independent predictor of recurrence beyond molecular risk [3,4]; nor do they justify any change in standard surveillance protocols. Any clinical actionability from this type of analysis requires external validation in stratified, prospective, molecularly annotated cohorts.

Exploratory analyses of reoperation and reirradiation did not identify consistent or robust associations with postoperative imaging features. While some variables showed non-significant trends in univariable analyses, these did not persist after adjustment in multivariable models. These findings likely reflect the limited number of events and reduced statistical power, and should be interpreted as hypothesis-generating rather than definitive, consistent with the multifactorial nature of treatment decision-making in glioma patients [6,15].

The results of this study are broadly consistent with previous literature emphasizing the importance of extent of resection and early imaging findings in glioma prognosis [4,7,8]. Most prior studies have, however, focused on immediate postoperative imaging or preoperative biomarkers, whereas fewer investigations have specifically addressed the prognostic role of the first post-treatment MRI [13,20]. Advanced imaging techniques—diffusion- and perfusion-weighted MRI, MR spectroscopy, and amino-acid PET—have been proposed to improve the differentiation of true progression from treatment-related changes [14,16,27,28], and quantitative diffusion measures such as the apparent diffusion coefficient (ADC) have shown prognostic value in dedicated cohorts [25]. In our cohort, DSC perfusion and diffusion sequences did not retain independent prognostic value in multivariable analyses; this likely reflects three factors of the present routine-care dataset—non-uniform acquisition of advanced sequences across the four-year accrual window, qualitative rather than quantitative assessment, and variable timing between completion of radiotherapy and first post-treatment MRI. These observations should not be read as evidence against the value of advanced MRI, but rather as a call for prospective protocol harmonization and dedicated quantitative analyses in future work.

This cohort intentionally reflects routine real-world practice in a tertiary neuro-oncology center and pools adult diffuse gliomas across the WHO 2021 spectrum, whose natural histories differ substantially. Pooling these entities therefore risks attenuating subtype-specific imaging–outcome associations: subgroup-specific gradients (e.g., the rapid recurrence dynamics of glioblastoma versus the indolent course of many IDH-mutant astrocytomas and oligodendrogliomas) may be averaged out in the present analysis. We chose this pooled framing for two pragmatic reasons—all subtypes are imaged with the same conventional MRI protocol and interpreted within a shared radiological vocabulary at our institution, consistent with structured surveillance frameworks for diffuse gliomas [29], and event counts within the lower-grade subgroups during the four-year accrual window were too small to support meaningful subtype-stratified multivariable modeling. Descriptive 1- and 2-year RFS stratified by histology, WHO grade, and IDH status are reported in Supplementary Table S7. Multidimensional prognostic frameworks such as the recently proposed HALLMOUNT score for grade 4 adult-type diffuse gliomas [30] represent a complementary axis of risk stratification that future imaging-based work may incorporate. Subtype-stratified analyses in larger, multicenter, molecularly annotated cohorts remain the priority next step.

Future studies should aim to validate these findings in larger, multicenter cohorts and to explore the integration of imaging features with molecular and genetic markers, as defined by the current WHO classification [3,4]. The incorporation of advanced imaging techniques and radiomics-based approaches may further improve the characterization of post-treatment changes and enhance early prediction of disease progression [15,20,21]. Furthermore, the increasing adoption of intraoperative resection-guidance technologies, such as 5-aminolevulinic acid (5-ALA) fluorescence-guided surgery [31], may shift the practical meaning of “ near-total resection” in routine practice and warrants integration into prospective imaging-prognostic frameworks. Future analyses with extended follow-up will additionally allow time-to-event modeling (Kaplan–Meier estimation, Cox proportional hazards) to characterize the temporal dynamics of recurrence in relation to imaging features.

### Limitations

This study has several limitations that should be considered when interpreting the findings. First, although IDH mutational status was available for 91.4% (127/139), ATRX for 78.4% (109/139), and KI-67 for 89.9% (125/139) of the cohort, full WHO 2021-aligned molecular profiling was not uniformly available: 1p/19q codeletion was available for 24.5% of patients, and other markers (TERT promoter mutation, EGFR amplification, p16/CDKN2A, CDKN2A/B homozygous deletion, H3 K27M) were available for fewer than 11%. MGMT promoter methylation status was not systematically captured in the institutional research database for the 2021–2024 window. The associations we report between conventional MRI features and recurrence-free outcomes therefore cannot be presumed to be independent of the underlying molecular risk profile. We have provided the available molecular distributions descriptively in the new Supplementary Table S6 and stratified descriptive 1- and 2-year RFS by IDH status (alongside histology and WHO grade) in Supplementary Table S7. Integrated clinico-radio-molecular profiling, with prospectively harmonized capture of IDH, MGMT, 1p/19q, ATRX, TERT, and CDKN2A/B in all patients, is the priority direction for future work.

Second, the cohort pools biologically and clinically distinct WHO 2021 entities (glioblastoma, IDH-mutant astrocytoma, oligodendroglioma), whose natural histories differ substantially. Although we have added descriptive subtype-stratified RFS in the Supplementary Materials (Table S7), event counts in the lower-grade subgroups precluded subtype-stratified multivariable modeling. The pooled estimates therefore represent a real-world average rather than subtype-specific risk.

Third, the operational definition of near-total resection (NTR; ≥80% volumetric removal) does not correspond to the strict RANO Resect Group definition of GTR (complete resection of the contrast-enhancing component) [24]. The 82.6% prevalence of residual tumor on the first post-treatment MRI is consistent with this near-total framing and is discussed in Section 3.1.

Fourth, the timing of the first post-treatment MRI was non-uniform across the cohort, reflecting the composite definition adopted (post-surgery-only vs. post-radiotherapy or chemoradiotherapy). The reported mean interval of approximately 120 days is an estimate from chart review rather than a calculated median with interquartile range. This timing variability is also entangled with the patient’s treatment pathway and could not be straightforwardly removed by adjustment.

Fifth, imaging interpretations were issued by neuroradiologists with full access to clinical and surgical history during routine reporting; formal blinding to outcome was not feasible. Because recurrence ascertainment relied on multidisciplinary review of the same imaging stream, the study is subject to both observer bias and incorporation bias. Advanced sequences (DWI, DSC-perfusion) were available only in a subset of patients with non-uniform protocols, and rCBV was assessed qualitatively rather than via quantitative analysis; this limits the statistical power and the interpretability of the perfusion findings.

Sixth, the analysis used binary outcome measures at fixed 1- and 2-year time points rather than time-to-event modeling. As justified in Section 2.5, this choice reflects the heterogeneity of follow-up windows, the imprecision of exact recurrence timing in the retrospective record, the unlikely validity of the proportional-hazards assumption across pooled WHO 2021 entities, and the exploratory framing of the study. Kaplan–Meier and Cox modeling are planned for future prospective work with extended and harmonized follow-up.

Seventh, the requirement of an available first post-treatment MRI led to the exclusion of 47% of initially screened patients (134/283), introducing a survivorship/selection element into the analytic cohort that we acknowledge explicitly. Recurrence adjudication, while combining imaging, clinical, and where available histopathological criteria reviewed by the multidisciplinary team, cannot fully exclude pseudoprogression as a contributor to apparent residual or recurrent disease in some cases. Finally, the multivariable models for the exploratory reoperation and re-irradiation endpoints (*n* = 44 and *n* = 41 respectively) have a low events-per-variable ratio and therefore carry a non-trivial risk of overfitting; the corresponding findings are reported as hypothesis-generating only.

## 5. Conclusions

In this study, some post-treatment MRI features showed associations with recurrence-free outcomes that warrant further evaluation in patients with diffuse gliomas after near-total resection. Among the evaluated imaging variables, the presence of residual tumor on the first post-treatment MRI was the most consistent imaging correlate of reduced RFS at both one and two years.

Structural MRI features evaluated (contrast enhancement of the residual tumor, diffusion restriction, postoperative hemorrhage, peritumoral edema, midline shift, post-operative infarction, and qualitative rCBV trend on DSC perfusion) did not retain independent associations with recurrence-free survival after adjustment, although several showed non-significant trends in the univariable analyses.

Exploratory analyses of reoperation and reirradiation, performed in smaller subsets with low events-per-variable ratios, did not identify consistent associations with first post-treatment MRI features. These exploratory analyses are reported as hypothesis-generating only and should not be interpreted as independent predictors.

Overall, the present results support the careful description of residual disease on the first post-treatment MRI in routine clinical reporting and may inform follow-up intensity at the individual-patient level. They do not, however, demonstrate that conventional first post-treatment MRI features identify independent predictors of recurrence beyond molecular risk, nor do they justify changes to standard surveillance protocols. External validation in stratified, prospective, molecularly annotated cohorts is required before any clinical actionability can be claimed from imaging features alone, and integration with molecular profiling and time-to-event modeling represents the priority direction for future work.

## Figures and Tables

**Table 1:** Baseline clinical and imaging characteristics of the study cohort (*n* = 139).

Variable	*n* (%), Excluding Missing Data
Sex	
Male	62 (44.6)
Female	77 (55.4)
**Histological diagnosis**	
Glioblastoma (GBM)	80 (57.6)
Astrocytoma	42 (30.2)
Oligodendroglioma	17 (12.2)
**WHO tumor grade**	
Grade 4	86 (61.9)
Grade 3	21 (15.1)
Grade 2	32 (23.0)
**Postoperative Karnofsky Performance Status**	
<90	20 (14.9)
90	44 (32.8)
100	70 (52.2)
**Tumor focality**	
Unifocal	125 (89.9)
Multifocal	14 (10.1)
**Tumor laterality**	
Left	70 (52.2)
Right	64 (47.8)
**Post-treatment residual tumor**	
Absent	24 (17.4)
Present	114 (82.6)
**Residual tumor contrast enhancement**	
Absent	31 (22.8)
Present	105 (77.2)
**Diffusion restriction**	
Absent	82 (65.6)
Present	43 (34.4)
**Post-treatment infarction**	
Absent	124 (91.2)
Present	12 (8.8)
**Post-treatment hemorrhage**	
Absent	14 (10.2)
Present	123 (89.8)
**Edema**	
Absent	4 (2.9)
Present	132 (97.1)
**Midline shift**	
Absent	106 (77.4)
Present	31 (22.6)

Values are presented as absolute numbers and percentages. Full descriptive statistics of all collected variables are provided in Supplementary Table S1.

**Table 2:** Univariable and multivariable logistic regression analysis for predictors of 1-year RFS after NTR.

Recurrence-Free Survival at 1 Year				
	*p* (Univariable)	OR (95% CI)	*p* (Multivariable)	aOR (95%)
**Postoperative KPS**				
lower	-	1.00	-	1.00
90	0.265	2.00 (0.59–6.76)	0.308	2.10 (0.50–8.79)
100	0.016	4.16 (1.30–13.36)	0.047	4.18 (1.01–17.18)
**Postoperative Residual Tumor**				
0	-	1.00	-	1.00
1	0.042	0.20 (0.04–0.94)	0.054	0.10 (0.01–1.04)
**Location of Residual Tumor**				
adjacent cortex	-	1.00		
deep margin	0.532	1.50 (0.42–5.35)		
cavity wall	0.436	1.52 (0.53–4.31)		
multifocal	-	-		
**Residual Tumor Contrast Enhancement**				
0	-	1.00	-	1.00
1	0.028	0.18 (0.04–0.83)	0.989	1.01 (0.14–7.03)
**Relative Cerebral Blood Volume (rCBV) Trend**				
highly increased	-	1.00		
increased	0.934	1.07 (0.23–4.89)		
normal	0.161	2.44 (0.70–8.53)		
decreased	0.167	2.67 (0.66–10.70)		
**Diffusion Restriction**				
0	-	1.00		
1	0.807	0.88 (0.34–2.29)		
**Postoperative Infarction**				
0	-	1.00		
1	0.839	0.86 (0.20–3.58)		
**Postoperative Hemorrhage**				
0	-	1.00		
1	0.325	0.45 (0.09–2.18)		
**Midline Shift**				
0	-	1.00	-	1.00
1	0.029	0.31 (0.11–0.89)	0.149	0.36 (0.09–1.44)

OR—odds ratio; aOR—adjusted odds ratio; CI—confidence interval; KPS—Karnofsky Performance Status. A dash (-) denotes the reference category (OR = 1.00), a variable not included in multivariable analysis, or a non-estimable category. Full regression models including all evaluated imaging variables are provided in Supplementary Table S2.

**Table 3:** Univariable and multivariable logistic regression analysis for predictors of 2-year RFS after NTR.

Recurrence-Free Survival at 2 Years				
	*p* (Univariable)	OR (95% CI)	*p* (Multivariable)	aOR (95%)
**Postoperative KPS**				
lower	-	1.00	-	1.00
90	0.191	2.25 (0.66–7.59)	0.208	2.56 (0.59–11.13)
100	0.008	4.84 (1.51–15.48)	0.029	4.94 (1.17–20.79)
**Postoperative Residual Tumor**				
0	-	1.00	-	1.00
1	0.026	0.17 (0.03–0.81)	0.043	0.09 (0.009–0.92)
**Location of Residual Tumor**				
adjacent cortex	-	1.00		
deep margin	0.780	1.19 (0.34–4.14)		
cavity wall	0.551	1.37 (0.48–3.87)		
multifocal	0.245	3.86 (0.39–37.58)		
**Residual Tumor Contrast Enhancement**				
0	-	1.00	-	1.00
1	0.016	0.15 (0.03–0.71)	0.958	0.95 (0.13–6.45)
**Relative Cerebral Blood Volume (rCBV) Trend**				
highly increased	-	1.00		
increased	0.699	1.35 (0.29–6.18)		
normal	0.077	3.09 (0.88–10.80)		
decreased	0.086	3.37 (0.84–13.55)		
**Diffusion Restriction**				
0	-	1.00		
1	0.932	1.04 (0.40–2.65)		
**Postoperative Infarction**				
0	-	1.00		
1	1.000	1.00 (0.24–4.13)		
**Postoperative Hemorrhage**				
0	-	1.00		
1	0.578	0.68 (0.17–2.65)		
**Midline Shift**				
0	-	1.00	-	1.00
1	0.061	0.37 (0.13–1.05)	0.284	0.47 (0.12–1.85)

OR—odds ratio; aOR—adjusted odds ratio; CI—confidence interval; KPS—Karnofsky Performance Status. A dash (-) denotes the reference category (OR = 1.00), a variable not included in multivariable analysis, or a non-estimable category. Full regression analyses including all evaluated imaging variables are presented in Supplementary Table S3.

## Data Availability

The data presented in this study are available on reasonable request from the corresponding author. The data are not publicly available due to privacy and ethical restrictions related to patient confidentiality.

## Supplementary Materials

The following supporting information can be downloaded at: https://www.mdpi.com/article/10.3390/medicina62061136/s1. Table S1: Detailed descriptive statistics of the study cohort; Table S2: Univariable and multivariable logistic regression analysis for recurrence-free survival longer than 1 year after near-total resection (RFS >1 year after NTR); Table S3: Univariable and multivariable logistic regression analysis for recurrence-free survival longer than 2 years after near-total resection (RFS >2 years after NTR); Table S4: Univariable and multivariable logistic regression analysis for postoperative reirradiation; Table S5: Univariable and multivariable logistic regression analysis for reoperation; Table S6: Availability and distribution of molecular markers in the analytic cohort (*n* = 139); Table S6.1: IDH status by histology and WHO 2021 grade (within *n* = 127 IDH-known subset); Table S7: Descriptive 1- and 2-year recurrence-free survival stratified by histology, WHO 2021 grade, and IDH status.

## Abbreviations

ADC	Apparent Diffusion Coefficient
aOR	Adjusted Odds Ratio
AIC	Akaike Information Criterion
ASTRO	Astrocytoma
BBB	Blood–Brain Barrier
CI	Confidence Interval
CNS	Central Nervous System
CT	Computed Tomography
DCE	Dynamic Contrast-Enhanced (MRI)
DSC	Dynamic Susceptibility Contrast (MRI)
DWI	Diffusion-Weighted Imaging
FLAIR	Fluid-Attenuated Inversion Recovery
GBM	Glioblastoma Multiforme
GTR	Gross Total Resection
IDH	Isocitrate Dehydrogenase
KPS	Karnofsky Performance Status
McF R^2^	McFadden’s Pseudo-R^2^
MGMT	O^6^-Methylguanine-DNA Methyltransferase
MRI	Magnetic Resonance Imaging
NTR	Near-total Resection
OLIGO	Oligodendroglioma
OR	Odds Ratio
PET	Positron Emission Tomography
PWI	Perfusion-Weighted Imaging
RANO	Response Assessment in Neuro-Oncology
rCBV	Relative Cerebral Blood Volume
RFS	Recurrence-Free Survival
STROBE	Strengthening the Reporting of Observational Studies in Epidemiology
SWI	Susceptibility-Weighted Imaging
TERT	Telomerase Reverse Transcriptase
WHO	World Health Organization

## References

[1] Ostrom Q.T., Shoaf M.L., Cioffi G., Waite K., Kruchko C., Wen P.Y., Brat D.J., Barnholtz-Sloan J.S., Iorgulescu J.B. (2023). National-level overall survival patterns for molecularly-defined diffuse glioma types in the United States. Neuro-Oncology.

[2] Weller M., van den Bent M., Preusser M., Le Rhun E., Tonn J.C., Minniti G., Bendszus M., Balana C., Chinot O., Dirven L. (2021). EANO guidelines on the diagnosis and treatment of diffuse gliomas of adulthood. Nat. Rev. Clin. Oncol..

[3] Louis D.N., Perry A., Wesseling P., Brat D.J., Cree I.A., Figarella-Branger D., Hawkins C., Ng H.K., Pfister S.M., Reifenberger G. (2021). The 2021 WHO Classification of Tumors of the Central Nervous System: A summary. Neuro-Oncology.

[4] Papacocea S.I., Vrinceanu D., Dumitru M., Manole F., Serboiu C., Papacocea M.T. (2024). Molecular Profile as an Outcome Predictor in Glioblastoma along with MRI Features and Surgical Resection: A Scoping Review. Int. J. Mol. Sci..

[5] Scheu K., Vandieken E., Hense K., Rosengarth K., Haedenkamp T., Lenglinger M., Bumes E., Linker R., Proescholdt M., Schmidt N.O. (2025). MRI-defined patterns of infiltration and outcome in patients with glioblastoma. Neuro-Oncol. Adv..

[6] Almenawer S.A., Badhiwala J.H., Alhazzani W., Greenspoon J., Farrokhyar F., Yarascavitch B., Algird A., Kachur E., Cenic A., Sharieff W. (2015). Biopsy versus partial versus gross total resection in older patients with high-grade glioma: A systematic review and meta-analysis. Neuro-Oncology.

[7] Brown T.J., Brennan M.C., Li M., Church E.W., Brandmeir N.J., Rakszawski K.L., Patel A.S., Rizk E.B., Suki D., Sawaya R. (2016). Association of the Extent of Resection with Survival in Glioblastoma: A Systematic Review and Meta-analysis. JAMA Oncol..

[8] Smets T., Lawson T.M., Grandin C., Jankovski A., Raftopoulos C. (2013). Immediate post-operative MRI suggestive of the site and timing of glioblastoma recurrence after gross total resection: A retrospective longitudinal preliminary study. Eur. Radiol..

[9] Faustino A.C., Viani G.A., Hamamura A.C. (2020). Patterns of recurrence and outcomes of glioblastoma multiforme treated with chemoradiation and adjuvant temozolomide. Clinics.

[10] Wang Z., Wang L., Wang Y. (2025). Radiomics in glioma: Emerging trends and challenges. Ann. Clin. Transl. Neurol..

[11] Chang S.M., Wen P.Y., Vogelbaum M.A., Macdonald D.R., van den Bent M.J. (2015). Response Assessment in Neuro-Oncology (RANO): More than imaging criteria for malignant glioma. Neuro-Oncol. Pract..

[12] Sanvito F., Castellano A., Cloughesy T.F., Wen P.Y., Ellingson B.M. (2024). RANO 2.0 criteria: Concepts applicable to the neuroradiologist’s clinical practice. Curr. Opin. Oncol..

[13] Rykkje A.M., Larsen V.A., Skjøth-Rasmussen J., Nielsen M.B., Carlsen J.F., Hansen A.E. (2023). Timing of Early Postoperative MRI following Primary Glioblastoma Surgery—A Retrospective Study of Contrast Enhancements in 311 Patients. Diagnostics.

[14] Masch W.R., Wang P.I., Chenevert T.L., Junck L., Tsien C., Heth J.A., Sundgren P.C. (2016). Comparison of Diffusion tensor imaging and magnetic resonance perfusion imaging in differentiating recurrent brain neoplasm from radiation necrosis. Acad. Radiol..

[15] Stetter I., Werner J.M., Wollring M., Ceccon G., Ciantar K.G., Stoffels G., Mottaghy F.M., Fink G.R., Langen K.-J., Lohmann P. (2025). Prediction of progression-free and overall survival following temozolomide chemoradiation using FET PET-based parameters including radiomics in patients with glioblastoma. Neuro-Oncol. Adv..

[16] Yang I., Huh N.G., Smith Z.A., Han S.J., Parsa A.T. (2010). Distinguishing glioma recurrence from treatment effect after radiochemotherapy and immunotherapy. Neurosurg. Clin. N. Am..

[17] Hoosemans L.J.N., Panayotopoulos L.F., van Kuijk S.M.J., Verduin M., Anten M.H.M.E., Pasmans R.C.O.S., Schellekens J.M., Huijs S.M.H., Verhoeff S.R., van den Berkmortel F.W.P.J. (2025). Clinical predictors of pseudoprogression in glioblastoma: A retrospective cohort analysis. J. Neurooncol.

[18] Herings S.D.A., van den Elshout R., de Wit R., Mannil M., Ravesloot C., Scheenen T.W.J., Arens A., van der Kolk A., Meijer F.J.A., Henssen D.J.H.A. (2024). How to evaluate perfusion imaging in post-treatment glioma: A comparison of three different analysis methods. Neuroradiology.

[19] Verma N., Cowperthwaite M.C., Burnett M.G., Markey M.K. (2013). Differentiating tumor recurrence from treatment necrosis: A review of neuro-oncologic imaging strategies. Neuro-Oncology.

[20] Li J., Xu Q., Fan X., Cheng X., Tao J., Lu H., Lin Q., Zhang J., Qian J. (2026). Multi-Sequence MRI radiomics model for discrimination of recurrence and pseudoprogression in gliomas. Eur. J. Radiol..

[21] Zhu Y., Gong Y., Xu W., Sun X., Jiang G., Qiu L., Shi K., Wu M., Fei Y., Yuan J. (2026). Development of a Prediction Model for Progression Risk in High-Grade Gliomas Based on Habitat Radiomics and Pathomics. Ann. Clin. Transl. Neurol..

[22] von Elm E., Altman D.G., Egger M., Pocock S.J., Gøtzsche P.C., Vandenbroucke J.P., STROBE Initiative (2008). The Strengthening the Reporting of Observational Studies in Epidemiology (STROBE) statement: Guidelines for reporting observational studies. J. Clin. Epidemiol..

[23] World Medical Association (2013). World Medical Association Declaration of Helsinki: Ethical principles for medical research involving human subjects. JAMA.

[24] Karschnia P., Dono A., Young J.S., Juenger S.T., Teske N., Häni L., Sciortino T., Mau C.Y., Bruno F., Nunez L. (2023). Prognostic evaluation of re-resection for recurrent glioblastoma using the novel RANO classification for extent of resection: A report of the RANO resect group. Neuro-Oncology.

[25] Bušić M., Rumboldt Z., Čerina D., Bušić Ž., Dolić K. (2024). Prognostic Value of Apparent Diffusion Coefficient (ADC) in Patients with Diffuse Gliomas. Cancers.

[26] Sidibe I., Tensaouti F., Roques M., Cohen-Jonathan-Moyal E., Laprie A. (2022). Pseudoprogression in Glioblastoma: Role of Metabolic and Functional MRI-Systematic Review. Biomedicines.

[27] Jenkinson M.D., Du Plessis D.G., Walker C., Smith T.S. (2007). Advanced MRI in the management of adult gliomas. Br. J. Neurosurg..

[28] Guarnera A., Ius T., Romano A., Bagatto D., Denaro L., Aiudi D., Iacoangeli M., Palmieri M., Frati A., Santoro A. (2025). Advanced MRI, Radiomics and Radiogenomics in Unravelling Incidental Glioma Grading and Genetic Status: Where Are We?. Medicina.

[29] Parillo M., Quattrocchi C.C. (2024). Brain Tumor Reporting and Data System (BT-RADS) for the Surveillance of Adult-Type Diffuse Gliomas after Surgery. Surgeries.

[30] Unlu A., Aydin A.A., Ozturk B., Turk C.C., Yildiz M. (2025). The HALLMOUNT Score: Development of a Novel Multidimensional Prognostic Model for Solid Tumors, with Initial Clinical Application in Grade 4 Adult-Type Diffuse Gliomas. Medicina.

[31] Ryskeldiyev N., Moldabekov A., Berdibayeva D., Maidan A., Tursynbekov T., Davletov D., Tleubergenov M., Kabykenova A., Kerimbayeva D., Doskaliyev A. (2025). Comparative Analysis of Clinical Outcomes in High-Grade Glioma Patients: 5-ALA Fluorescence-Guided Surgery vs. Conventional White-Light Resection. Cancers.

