# Supplementary material for: Post-Treatment MRI Features on First Follow-Up Imaging in Diffuse Gliomas After Near-Total Resection: A Real-World Exploratory Cohort Study
Source: Medicina (Kaunas). 2026 Jun 10;62(6):1136. doi: 10.3390/medicina62061136 (PMC13303984; doi:10.3390/medicina62061136)
Supplement: Supplementary file 1 [file medicina-62-01136-s001.zip › medicina-4317429-supplementary.pdf]

medicina

MDPI

Supplementary Material

Supplementary Table S1. Detailed descriptive statistics of the study cohort.

Variable	n	%
**Recurrence-Free Survival >1 Year After NTR**		
0	31.0	27.7
1	81.0	72.3
**Recurrence-Free Survival >2 Years After NTR**		
0	34.0	30.4
1	78.0	69.6
**Reoperation**		
0	34.0	70.8
1	14.0	29.2
**Postoperative Re-irradiation**		
0	30.0	62.5
1	18.0	37.5
**Sex**		
M	62.0	44.6
F	77.0	55.4
**Histological Diagnosis**		
GBM	80.0	57.6
ASTRO	42.0	30.2
OLIGO	17.0	12.2
**WHO Tumor Grade**		
G4	86.0	61.9
G2	32.0	23.0
G3	21.0	15.1
**Initial Clinical Presentation**		
other	57.0	41.0
language deficit	24.0	17.3
seizures	58.0	41.7
**Postoperative KPS**		
lower	20.0	14.9
90	44.0	32.8
100	70.0	52.2
**Tumor Focality**		
0	125.0	89.9
1	14.0	10.1
**Tumor Laterality**		
L	70.0	52.2
R	64.0	47.8
**Tumor Location**		

other	29.0	20.9
Fr	70.0	50.4
Tm	40.0	28.8
**Postoperative Residual Tumor**		
0	24.0	17.4
1	114.0	82.6
**Location of Residual Tumor**		
adjacent cortex	32.0	26.0
deep margin	27.0	22.0
cavity wall	51.0	41.5
multifocal	13.0	10.6
**Residual Tumor Contrast Enhancement**		
0	31.0	22.8
1	105.0	77.2
**Relative Cerebral Blood Volume (rCBV) Trend**		
highly increased	19.0	19.6
increased	15.0	15.5
normal	41.0	42.3
decreased	22.0	22.7
**Diffusion Restriction**		
0	82.0	65.6
1	43.0	34.4
**Postoperative Infarction**		
0	124.0	91.2
1	12.0	8.8
**Postoperative Hemorrhage**		
0	14.0	10.2
1	123.0	89.8
**Edema**		
0	4.0	2.9
1	132.0	97.1
**Midline Shift**		
0	106.0	77.4
1	31.0	22.6

Absolute and relative frequencies of collected qualitative variables in our sample (n=139). This table presents the full descriptive distribution of all clinical and imaging variables included in the study cohort. Values are expressed as absolute numbers (n) and percentages (%).

Supplementary Table S2. Univariable and multivariable logistic regression analysis for recurrence-free survival longer than 1 year after near-total resection (RFS >1 year after NTR).

**Recurrence-Free 1. Year After NTR**				
	**p (univariable)**	**OR (95% CI)**	**p (multivariable)**	**aOR (95%)**
**Sex**				
M	-	1.00	-	1.00

F	0.371	1.47 (0.63-3.42)	0.288	0.56 (0.19-1.63)
**Histological Diagnosis**				
GBM	-	1.00	-	1.00
ASTRO	0.03	3.07 (1.11-8.48)	0.916	1.16 (0.07-19.07)
OLIGO	-	-		
**WHO Tumor Grade**				
G4	-	1.00	-	1.00
G2	0.017	4.86 (1.32-17.83)	0.425	3.90 (0.13-111.14)
G3	0.033	5.39 (1.14-25.28)	0.905	0.83 (0.03-17.76)
**Age at Diagnosis**	0.015	0.963 (0.93-0.99)	0.421	0.97 (0.93-1.03)
**Initial Clinical Presentation**				
other	-	1.00		
language deficit	0.834	1.13 (0.36-3.52)		
seizures	0.244	1.74 (0.68-4.43)		
**Postoperative KPS**				
lower	-	1.00	-	1.00
90	0.265	2.00 (0.59-6.76)	0.308	2.10 (0.50-8.79)
100	0.016	4.16 (1.30-13.36)	0.047	4.18 (1.01-17.18)
**Tumor Focality**				
0	-	1.00		
1	0.751	0.75 (0.13-4.34)		
**Tumor Laterality**				
L	-	1.00		
R	0.571	1.28 (0.54-3.03)		
**Tumor Location**				
other	-	1.00		
Fr	0.827	1.13 (0.39-3.25)		
Tm	0.415	1.67 (0.48-5.69)		
**Postoperative Residual Tumor**				
0	-	1.00	-	1.00
1	0.042	0.20 (0.04-0.94)	0.054	0.10 (0.01-1.04)
**Postoperative Residual Tumor Volume (cm^3)**	0.814	1.01 (0.95-1.05)		
**Location of Residual Tumor**				
adjacent cortex	-	1.00		
deep margin	0.532	1.50 (0.42-5.35)		
cavity wall	0.436	1.52 (0.53-4.31)		
multifocal	-	-		
**Residual Tumor Contrast Enhancement**				
0	-	1.00	-	1.00
1	0.028	0.18 (0.04-0.83)	0.989	1.01 (0.14-7.03)
**Relative Cerebral Blood Volume (rCBV) Trend**				
highly increased	-	1.00		
increased	0.934	1.07 (0.23-4.89)		
normal	0.161	2.44 (0.70-8.53)		
decreased	0.167	2.67 (0.66-10.70)		
**Diffusion Restriction**				
0	-	1.00		
1	0.807	0.88 (0.34-2.29)		

**Postoperative Infarction**				
0	-	1.00		
1	0.839	0.86 (0.20-3.58)		
**Postoperative Hemorrhage**				
0	-	1.00		
1	0.325	0.45 (0.09-2.18)		
**Midline Shift**				
0	-	1.00	-	1.00
1	0.029	0.31 (0.11-0.89)	0.149	0.36 (0.09-1.44)
			**AIC**	113
			**McF r^2**	0.213
			**overall p**	0.006
			**n**	93

Univariable and multivariable logistic regression analyses were performed to identify factors associated with recurrence-free survival longer than 1 year after NTR. Variables with p < 0.20 in the univariable analysis, as well as sex (considered a clinically relevant variable), were included in the multivariable model. Categories showing quasi-complete or complete separation were excluded from the analysis. OR = odds ratio; aOR = adjusted odds ratio; CI = confidence interval; AIC = Akaike Information Criterion; McFadden's R^2 = McFadden's pseudo-coefficient of determination; n = number of cases included in the model.

Supplementary Table S3. Univariable and multivariable logistic regression analysis for recurrence-free survival longer than 2 years after near-total resection (RFS >2 year after NTR).

Recurrence-Free Survival >2 Years After NTR (Yes vs No)

	p (univariable)	OR (95% CI)	p (multivariable)	aOR (95%)
**Sex**				
M	-	1.00	-	1.00
F	0.541	0.77 (0.34-1.75)	0.486	0.69 (0.24-1.95)
**Histological Diagnosis**				
GBM	-	1.00	-	1.00
ASTRO	0.011	3.73 (1.35-10.27)	0.576	2.25 (0.13-36.63)
OLIGO	-	-		
**WHO Tumor Grade**				
G4	-	1.00	-	1.00
G2	0.008	5.85 (1.60-21.39)	0.445	3.68 (0.12-104.93)
G3	0.018	6.49 (1.38-30.34)	0.834	0.72 (0.03-15.64)
**Age at Diagnosis**	0.014	0.96 (0.93-0.99)	0.763	0.99 (0.94-1.04)
**Initial Clinical Presentation**				
other	-	1.00		
language deficit	0.879	1.09 (0.36-3.26)		
seizures	0.112	2.11 (0.84-5.30)		
**Postoperative KPS**				
lower	-	1.00	-	1.00
	900.191	2.25 (0.66-7.59)	0.208	2.56 (0.59-11.13)

	1000.008	4.84 (1.51-15.48)	0.029	4.94 (1.17-20.79)
**Tumor Focality**				
	0-	1.00		
	10.295	0.41 (0.07-2.16)		
**Tumor Laterality**				
L	-	1.00		
R	0.346	1.50 (0.64-3.51)		
**Tumor Location**				
other	-	1.00		
Fr	0.544	1.38 (0.48-3.89)		
Tm	0.523	1.46 (0.45-4.66)		
**Postoperative Residual Tumor**				
	0-	1.00	-	1.00
	10.026	0.17 (0.03-0.81)	0.043	0.09 (0.009-0.92)
**Postoperative Residual Tumor Volume (cm³)**	0.754	1.01 (0.96-1.05)		
**Location of Residual Tumor**				
adjacent cortex	-	1.00		
deep margin	0.780	1.19 (0.34-4.14)		
cavity wall	0.551	1.37 (0.48-3.87)		
multifocal	0.245	3.86 (0.39-37.58)		
**Residual Tumor Contrast Enhancement**				
	0-	1.00	-	1.00
	10.016	0.15 (0.03-0.71)	0.958	0.95 (0.13-6.45)
**Relative Cerebral Blood Volume (rCBV) Trend**				
highly increased	-	1.00		
increased	0.699	1.35 (0.29-6.18)		
normal	0.077	3.09 (0.88-10.80)		
decreased	0.086	3.37 (0.84-13.55)		
**Diffusion Restriction**				
	0-	1.00		
	10.932	1.04 (0.40-2.65)		
**Postoperative Infarction**				
	0-	1.00		
	11,000	1.00 (0.24-4.13)		
**Postoperative Hemorrhage**				
	0-	1.00		
	10.578	0.68 (0.17-2.65)		
**Midline Shift**				
	0-	1.00	-	1.00
	10.061	0.37 (0.13-1.05)	0.284	0.47 (0.12-1.85)
			**AIC**	114
			**McF r^2**	0.229
			**overall p**	0.002
			**n**	93

Univariable and multivariable logistic regression analyses were performed to identify factors associated with recurrence-free survival longer than 2 year after NTR. Categories exhibiting quasi- or complete separation were dropped from the analysis. Variables with p<0.2 in the univariable

analysis, and the clinically variable sex, were used in building the multivariable model. AIC, McFadden's R^2, and overall model p value were calculated for the multivariable model. OR=odds ratio, aOR=adjusted odds ratio, AIC=Akaike Information Criterion, n=sample size used for model estimation.

Supplementary Table S4. Univariable and multivariable logistic regression analysis for postoperative reirradiation.

Postoperative Re-irradiation				
	**p (univariable)**	**OR (95% CI)**	**p (multivariable)**	**aOR (95%)**
**Sex**				
M	-	1.00	-	1.00
F	0.366	1.75 (0.52-5.89)	0.448	0.447 (0.04-3.56)
**Histological Diagnosis**				
GBM	-	1.00		
ASTRO	0.007	21.77 (2.35-201.68)		
OLIGO	0.154	6.22 (0.50-76.96)		
**WHO Tumor Grade**				
G4	-	1.00		
G2	-	-		
G3				
**Age at Diagnosis**	<0.001	0.88 (0.82-0.95)	0.029	0.88 (0.79-0.98)
**Initial Clinical Presentation**				
other	-	1.00		
language deficit	0.900	0.90 (0.17-4.64)		
seizures	0.674	0.75 (0.19-2.86)		
**Postoperative KPS**				
lower	-	1.00	-	1.00
	900.775	0.76 (0.12-4.65)	0.947	0.92 (0.08-10.21)
	1000.040	5.55 (1.07-28.63)	0.554	1.97 (0.20-18.89)
**Tumor Focality**				
	0-	1.00		
	10.429	0.55 (0.12-2.42)		
**Tumor Laterality**				
L	-	1.00	-	1.00
R	0.122	2.71 (0.76-9.63)	0.336	2.49 (0.38-16.13)
**Tumor Location**				
other	-	1.00	-	1.00
Fr	0.247	2.54 (0.52-12.37)	0.687	0.50 (0.01-14.16)
Tm	0.568	0.58 (0.09-3.72)	0.442	0.30 (0.01-6.37)
**Postoperative Residual Tumor**				
	0-	1.00		
	10.308	0.27 (0.02-3.28)		
**Postoperative Residual Tumor Volume (cm^3)**	0.430	0.98 (0.95-1.02)		
**Location of Residual Tumor**				
adjacent cortex	-	1.00		
deep margin	0.673	1.45 (0.25-8.43)		

cavity wall	0.696	1.40 (0.25-7.58)		
multifocal	0.889	1.16 (0.13-10.22)		
**Residual Tumor Contrast Enhancement**				
	0-	1.00		
	10.681	0.55 (0.03-9.43)		
**Relative Cerebral Blood Volume (rCBV) Trend**				
highly increased	-	1.00		
increased	0.172	0.17 (0.01-2.12)		
normal	0.845	0.83 (0.13-5.17)		
decreased	0.615	1.66 (0.22-12.22)		
**Diffusion Restriction**				
	0-	1.00		
	10.813	1.18 (0.29-4.69)		
**Postoperative Infarction**				
	0-	1.00		
	10.609	0.54 (0.05-5.66)		
**Postoperative Hemorrhage**				
	0-	1.00		
	10.241	0.37 (0.07-1.93)		
**Midline Shift**				
	0-	1.00		
	10.645	0.75 (0.22-2.55)		
			**AIC**	49.1
			**McF r^2**	0.371
			**overall p**	0.007
			**n**	41

Univariable and multivariable logistic regression analyses were performed to identify factors associated with postoperative reirradiation. Categories exhibiting quasi- or complete separation were dropped from the analysis. Variables with p<0.2 in the univariable analysis, and the clinically variable sex, were used in building the multivariable model. AIC, McFadden's R^2, and overall model p value were calculated for the multivariable model. OR=odds ratio, aOR=adjusted odds ratio, AIC=Akaike Information Criterion, n=sample size used for model estimation.

Supplementary Table S5. Univariable and multivariable logistic regression analysis for reoperation.

Reoperation

	**p (univariable)**	**OR (95% CI)**	**p (multivariable)**	**aOR (95%)**
**Sex**				
M	-	1.00	-	1.00
F	0.180	2.50 (0.65-9.55)	0.683	1.45 (0.24-8.62)
**Histological Diagnosis**				
GBM	-	1.00	-	1.00
ASTRO	0.113	3.62 (0.73-17.80)	0.207	0.13 (0.005-3.04)
OLIGO	0.124	7.25 (0.58-90.54)	0.614	0.43 (0.01-10.73)

**WHO Tumor Grade**				
G4	-	1.00		
G2	0.283	2.50 (0.47-13.30)		
G3	-	-		
**Age at Diagnosis**	0.002	0.90 (0.84-0.96)	0.008	0.85 (0.75-0.95)
**Initial Clinical Presentation**				
other	-	1.00		
language deficit	0.232	0.25 (0.02-2.41)		
seizures	0.543	0.64 (0.15-2.64)		
**Postoperative KPS**				
lower	-	1.00		
	90-	-		
	1000.300	2.25 (0.48-10.41)		
**Tumor Focality**				
	0-	1.00		
	10.875	0.88 (0.19-3.98)		
**Tumor Laterality**				
L	-	1.00	-	1.00
R	0.185	2.45 (0.65-9.21)	0.183	3.35 (0.56-19.93)
**Tumor Location**				
other	-	1.00		
Fr	0.209	3.07 (0.53-17.80)		
Tm	0.658	0.61 (0.07-5.28)		
**Postoperative Residual Tumor**				
	0-	1.00		
	1-	-		
**Postoperative Residual Tumor Volume (cm³)**	0.247	1.008 (0.99-1.02)		
**Location of Residual Tumor**				
adjacent cortex	-	1.00		
deep margin	0.478	0.52 (0.08-3.12)		
cavity wall	0.598	0.63 (0.11-3.41)		
multifocal	0.587	1.75 (0.23-13.16)		
**Residual Tumor Contrast Enhancement**				
	0-	1.00		
	1-	-		
**Relative Cerebral Blood Volume (rCBV) Trend**				
highly increased	-	1.00		
increased	0.858	1.20 (0.16-8.80)		
normal	0.571	0.57 (0.07-4.64)		
decreased	0.488	2.00 (0.28-14.20)		
**Diffusion Restriction**				
	0-	1.00		
	10.235	2.32 (0.57-9.36)		
**Postoperative Infarction**				
	0-	1.00		
	1-	-		
**Postoperative Hemorrhage**				
	0-	1.00		

https://doi.org/10.3390/xxxxx

	10.443	0.52 (0.10-2.73)			
**Midline Shift**					
	0-	1.00			
	10.623	1.37 (0.38-4.90)			
			**AIC**	47.5	
			**McF r^2**	0.336	
			**overall p**	0.003	
			**n**		44

Univariable and multivariable logistic regression analyses were performed to identify factors associated with reoperation. Categories exhibiting quasi- or complete separation were dropped from the analysis. Variables with p<0.2 in the univariable analysis, and the clinically variable sex, were used in building the multivariable model. AIC, McFadden's R^2, and overall model p value were calculated for the multivariable model. OR=odds ratio, aOR=adjusted odds ratio, AIC=Akaike Information Criterion, n=sample size used for model estimation.

Supplementary Table S6. Availability and distribution of molecular markers in the analytic cohort (n = 139).

Marker	n with data (%)	Negative / wild-type	Positive / mutant	% positive
IDH (IDH1/IDH2 mutation)	127/139 (91.4)	73	54	42.5
KI-67 (proliferation index)	125/139 (89.9)	63	62	49.6
ATRX (loss of nuclear expression)	109/139 (78.4)	82	27	24.8
1p/19q codeletion	34/139 (24.5)	19	15	44.1
EGFR amplification	14/139 (10.1)	4	10	71.4
p16 / CDKN2A (loss)	10/139 (7.2)	6	4	40.0
TERT promoter mutation	5/139 (3.6)	3	2	40.0
CDKN2A/B homozygous deletion	5/139 (3.6)	4	1	20.0
H3 K27M	3/139 (2.2)	2	1	33.3

IDH = isocitrate dehydrogenase (mutation = 1, wild-type = 0); ATRX = alpha-thalassemia/mental retardation X-linked (loss of nuclear expression by IHC = 1; retained = 0); KI-67 proliferation index dichotomized at the institutional reporting threshold (≥20% = 1, <20% = 0); EGFR amplification by FISH or IHC; TERT promoter mutation by PCR/sequencing; CDKN2A/B homozygous deletion and H3 K27M assessed on histopathological–molecular grounds where clinically indicated. The selective availability of advanced molecular markers (1p/19q, EGFR, TERT, CDKN2A/B, H3 K27M) reflects the routine-care nature of this dataset and is acknowledged in the Limitations.

Supplementary Table S6.1. IDH status by histology and WHO 2021 grade (within n = 127 IDH-known subset).

Stratum	IDH-mutant	IDH-wildtype	Total (IDH-known)	% IDH-mutant
Histology				
Glioblastoma (GBM)	1	69	70	1.4

Stratum	IDH-mutant	IDH-wildtype	Total (IDH-known)	% IDH-mutant
Astrocytoma	37	4	41	90.2
Oligodendroglioma	16	0	16	100.0
WHO 2021 grade				
Grade 2	29	2	31	93.5
Grade 3	19	1	20	95.0
Grade 4	6	70	76	7.9
**Total (n = 127)**	**54**	**73**	**127**	**42.5**

Distribution of IDH status across histological subtypes and WHO 2021 grades in the IDH-known subset (n = 127). The pattern is consistent with the WHO 2021 framework: IDH-wildtype status predominates in glioblastoma and grade 4 tumors; IDH-mutant status predominates in astrocytoma, oligodendroglioma, and grade 2–3 tumors.

Supplementary Table S7. Descriptive 1- and 2-year recurrence-free survival stratified by histology, WHO 2021 grade, and IDH status.

Stratum	n (total)	n with 1y outcome	1y RFS, n (%)	1y recurrence	2y RFS, n (%)	2y recurrence
Histology						
Glioblastoma (GBM)	80	63	38 (60.3)	25	35 (55.6)	28
Astrocytoma	42	34	28 (82.4)	6	28 (82.4)	6
Oligodendroglioma	17	15	15 (100.0)	0	15 (100.0)	0
WHO 2021 grade						
Grade 2	32	26	23 (88.5)	3	23 (88.5)	3
Grade 3	21	19	17 (89.5)	2	17 (89.5)	2
Grade 4	86	67	41 (61.2)	26	38 (56.7)	29
IDH status (within n = 127 IDH-known)						
IDH-mutant	54	45	39 (86.7)	6	39 (86.7)	6
IDH-wildtype	73	59	37 (62.7)	22	35 (59.3)	24

Descriptive recurrence-free survival (RFS) at 1 and 2 years after near-total resection (NTR), stratified by histological subtype, WHO 2021 grade, and IDH status. RFS is reported as the proportion of patients without recurrence at the corresponding landmark, computed by complete-case analysis among patients with an ascertained outcome at that landmark.
